# N-acetylcysteine Protects against Apoptosis through Modulation of Group I Metabotropic Glutamate Receptor Activity

**DOI:** 10.1371/journal.pone.0032503

**Published:** 2012-03-19

**Authors:** Lili Sun, Li Gu, Shuting Wang, Jifang Yuan, Huimin Yang, Jiawei Zhu, Hong Zhang

**Affiliations:** 1 Department of Cell Biology, Key Laboratory for Neurodegenerative Disease, Ministry of Education, Capital Medical University, Beijing, China; 2 Department of Neurobiology, Key Laboratory for Neurodegenerative Disease, Ministry of Education, Capital Medical University, Beijing, China; University of Houston, United States of America

## Abstract

The activation of group I metabotropic glutamate receptor (group I mGlus) has been shown to produce neuroprotective or neurotoxic effects. In this study, we investigated the effects of N-acetylcysteine (NAC), a precursor of the antioxidant glutathione, on group I mGlus activation in apoptosis of glial C6 and MN9D cell lines, and a rat model of Parkinson's disease (PD). We demonstrated that NAC protected against apoptosis through modulation of group I mGlus activity. In glial C6 cells, NAC promoted phosphorylation of ERK induced by (s)-3,5- dihydroxy-phenylglycine (DHPG), an agonist of group I mGlus. NAC enhanced the group I mGlus-mediated protection from staurosporine (STS)-induced apoptosis following DHPG treatment. Moreover**,** in rotenone-treated MN9D cells and PD rat model, NAC protected against group I mGlus-induced toxicity by compromising the decrease in phosphorylation of ERK, phosphorylation or expression level of TH. Furthermore, the results showed that NAC prohibited the level of ROS and oxidation of cellular GSH/GSSG (E_h_) accompanied by activated group I mGlus in the experimental models. Our results suggest that NAC might act as a regulator of group I mGlus-mediated activities in both neuroprotection and neurotoxicity via reducing the oxidative stress, eventually to protect cell survival. The study also suggests that NAC might be a potential therapeutics targeting for group I mGlus activation in the treatment of PD.

## Introduction

Metabotropic glutamate receptors (mGlus) are G-protein-coupled receptors that can be classified into group I receptors (mGlu1 and mGlu5), groups II (mGlu2 and mGlu3) and III (mGlu4, mGlu6, mGlu7 and mGlu8), based on their signal transduction pathways and pharmacologic profiles. Increasing evidence has indicated roles for group I metabotropic glutamate receptors (group I mGlus) in a variety of disorders, including Parkinson's disease (PD), amyotrophic lateral sclerosis, epilepsy, stroke and Alzheimer's disease [1]. The activation of group I mGlus has been shown to produce neuroprotective or neurotoxic effects in cell viabilty [2]. It is therefore of considerable interest to investigate the regulation of group I mGlus in the context of neuroprotection in more details.

The activation of group I mGlus can be neuroprotective or neurotoxic effects depending on different stimuli or the molecular mechanism by which the signaling is achieved. Activation of group I mGlus can either exacerbate neuronal death induced by oxygen-glucose deprivation [3] or attenuate oligodendrocyte excitoxicity by inhibiting the accumulation of reactive oxygen species (ROS) and intracellular glutathione (GSH) loss [4]. Group I mGlus activation in neuroprotection initiates various intracellular signaling mechanisms, including extracellular signal-regulated kinase (ERK) [5]–[6]. Activation of the ERK pathway can be involved in neuroprotection [7]–[9], or neurotoxicity [10]–[12]. Moreover, activation of group I mGlus is also involved in apoptosis signaling. For example, DHPG elicited a significant increase in poly (ADP-ribose) polymerase (PARP) activity that was completely abolished by the administration of the mGlu1 antagonist 3-MATIDA and partially prevented by the mGlu5 antagonist MPEP [13].

Since activation of group I mGlus can be neuroprotective or neurotoxic, it is important to understand the mechanism responsible for modulation of the receptor activity in cell survival. Previous study has shown that the activation of mGlus protects nerve cells from oxidative stress [14]. We therefore wanted to test whether group I mGlus-mediated cell survival is regulated by NAC treatment. In the study, we investigated the effects of NAC on the activation of group I mGlus in glial C6 and MN9D cells, and in a rotenone-induced rat model of PD. Interestingly, we found that NAC can protect against cell apoptosis in both situations of neuroprotection and neurotoxicity through modulating group I mGlus-mediated ERK activity, suggesting that NAC might act as a regulator of group I mGlus-mediated activity for receptor to be neuroprotective.

## Results

### NAC enhanced phospho-ERK induced by activation of group I mGlus in glial C6 cells

**Figure 1:**
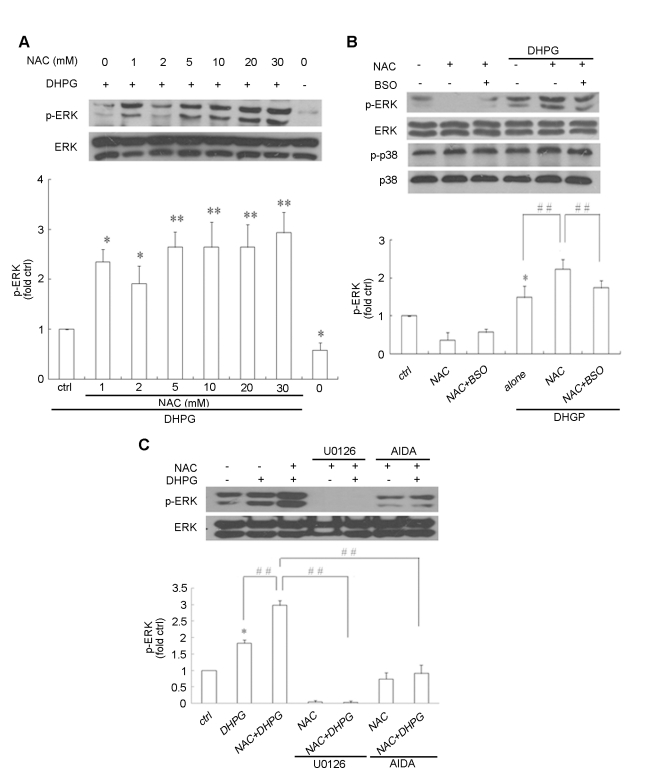
The effect of NAC on group I mGlus activation-mediated phospho-ERK, phospho-p38 in glial C6 cells. NAC was applied to C6 glial cells for 30 min, with or without pretreatment with 100 µM BSO for 12 h, followed by DHPG 100 µM treatment for 5 min. (A) Exposure to different concentrations of NAC enhanced DHPG-induced phospho-ERK. (B) BSO treatment partially reversed the effects of NAC on group I mGlus activation-mediated phospho-ERK, and neither NAC nor BSO had any significant effect on phospho-p38. (C) NAC upregulated phospho-ERK stimulated by DHPG was prevented by both group I mGlus antagonist AIDA (100 µM) and the MEK inhibitor U0126 (20 µM). Non-treated cells were used as controls in all panels. (A, B and C) Representative immunoblots are shown above the quantitative data for pERK1/2. *p<0.05 and **p<0.01 versus control; # p<0.05, # # p<0.01.

Recent studies showed that group I mGlus was expressed in C6 glial cells [15] and that activation of these receptors by DHPG led to phosphorylation of ERK, which was well characterized in the activity of group I mGlus [16]–[17]. To examine the effects of NAC on the activation of group I mGlus, we first investigated the effects of NAC on ERK phosphorylation in response to DHPG treatment in glial C6 cells. As shown in Fig. 1A, consistent with previous studies [16]–[17], exposure of cells to DHPG promoted phospho-ERK. NAC enhanced DHPG-induced phospho-ERK in a dose dependent manner, and reached maximal effect at 5 mM. This concentration was therefore used in subsequent experiments. To confirm the role of NAC, a precursor of glutathione (GSH), in the activation of ERK by group I mGlus, the selective GSH-depleting agent, buthionine sulfoximine (BSO) was introduced. The enhancement of ERK phosphorylation by NAC was partially reversed by BSO, indicating that GSH-controlled anti-oxidative stress was involved in the regulation of ERK phosphorylation by group I mGlus (Fig. 1B ). It has been shown that activation of group I mGlus was not involved in the p38 MAP kinase pathway [17]. Consistent with DHPG treatment in the report, neither NAC nor BSO affected the levels of phospho-p38 (Fig. 1B), suggesting that NAC might specifically enhanced DHPG-induced ERK phosphorylation. To examine the involvement of group I mGlus activation and the ERK pathway in the mechanism of NAC, (RS)-1-aminoindan-1,5-dicarboxylic acid (AIDA , an antagonist of group I mGlus) and U0126 (an inhibitor of MEK) were applied before DHPG application, both of which significantly attenuated the promotion of DHPG-induced ERK phosphorylation by NAC (Fig. 1C).

### NAC enhanced group I mGlus-mediated protection against STS toxicity in glial C6 cells

**Figure 2:**
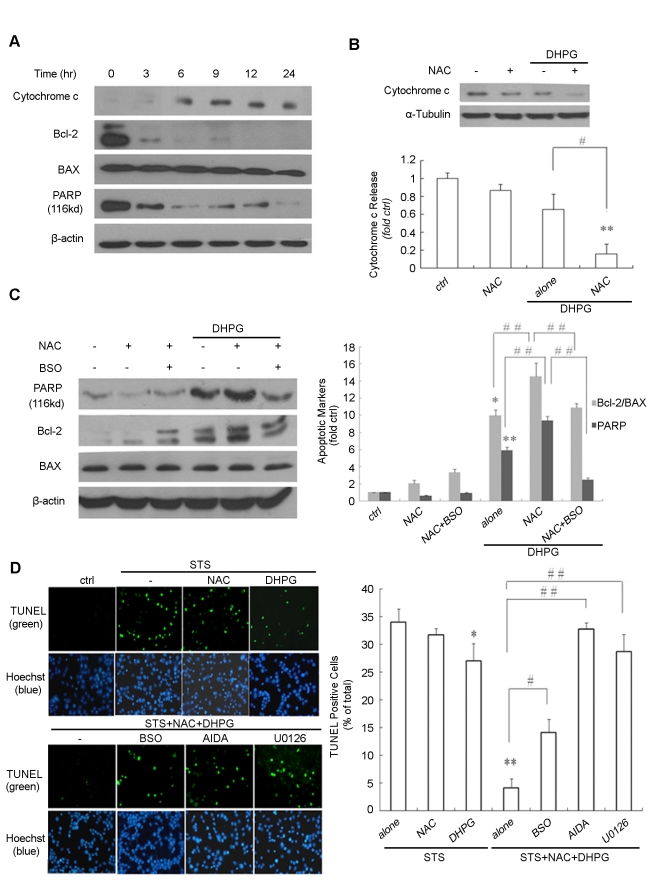
NAC enhanced group I mGlus-mediated protection against STS toxicity in glial C6 cells. (A) Cells were treated with 1 µM STS for the indicated time. STS treatment led to an increase in cytochrome c release into the cytosol and a fall in the Bcl-2/Bax ratio and PARP (116 kD) level in a time-dependent manner. (B) Cells were exposed to DHPG (100 µM, 30 min) after 5 mM NAC pretreatment, followed by STS exposure together with NAC and DHPG incubation for 9 h. Release of cytochrome c into the cytosol was partly inhibited by DHPG exposure, and further strengthened by NAC pretreatment. A representative immunoblot is shown above the quantitative data. (C) Cells were preincubated with DHPG (100 µM, 30 min) in the presence of either NAC or NAC/BSO, followed by STS exposure together with DHPG and NAC for 12 h. NAC promoted the DHPG-induced increase in the Bcl-2/Bax ratio and prevented the cleavage of PARP, which could be reversed by BSO pretreatment. A representative immunoblot is shown to the right of the quantitative data. (D) The effect of NAC on DHPG-mediated apoptosis was further examined in TUNEL staining experiments. Cells were exposed to NAC (5 mM ) or DHPG (100 µM, 30 min) after pretreatment with BSO (100 µM), the group I mGlus antagonist AIDA(100 µM) or the MEK inhibitor U0126 (20 mM), followed by STS exposure for 12 h. Magnification ×400 (n = 4–6 microscopic fields, 100–300 glial C6 cells per field) (left). Densitometric analysis of TUNEL-positive cells, expressed as a percentage of total cells (right). *p<0.05 and **p<0.01 versus control; #p<0.05, # #p<0.01.

Because NAC can affect group I mGlus-mediated ERK signaling in glial C6 cells, we further investigated its ability to modulate cell apoptosis by affecting group I mGlus activity. Cells were treated with the well-established stress agent, STS, which induces apoptosis via mitochondria-dependent pathways [18]–[19]. Cytochrome c release, Bcl-2/Bax ratio and cleavage of PARP were measured as markers of apoptosis. STS treatment led to a time-dependent increase in cytochrome c release into the cytosol, and reduction in the Bcl-2/Bax ratio and the level of uncleaved PARP (Fig. 2A). Release of cytochrome c into the cytosol was partly inhibited by DHPG exposure, and this effect was strengthened by pretreatment with NAC (Fig. 2B). Moreover, NAC promoted the DHPG-induced increase in the Bcl-2/Bax ratio and prevented the cleavage of PARP, which could be reversed by BSO pretreatment (Fig. 2C). We further tested the effect of caspase-3 in NAC protection against group I mGlus-mediated cellular apoptosis. Under the experimental conditions, pretreatment of Z-DEVD-FMK, a specific caspase-3 inhibitor, with DHPG application inhibited STS-induced cell death, suggesting that caspase-3 is involved in NAC protection against group I mGlus-mediated cellular apoptosis (Fig. S1). These results indicate that NAC enhanced the protective effects of activated group I mGlus against STS toxicity. The effects of NAC on DHPG-mediated apoptosis were further examined by TUNEL staining. As shown in Fig. 2D, STS exposure was accompanied by an increase in the number of TUNEL-positive, apoptotic cells, which was reduced by DHPG exposure accompanied by NAC treatment, compared with DHPG treatment alone. Pretreatment with BSO, the group I mGlus antagonist AIDA, or the MEK inhibitor U0126, reversed the effects of NAC on cell apoptosis with DHPG exposure. These results confirmed an enhanced apoptosis-protective effect of NAC against STS toxicity via group I mGlus-activated ERK pathway.

### NAC protects against group I mGlus-induced toxicity in rotenone-treated MN9D cells and an animal model of PD

Activation of mGlus plays a pivotal role in PD [20]–[21], while mitochondrial dysfunction and oxidative stress have also been shown to be involved [22]. Thus, we wanted to test the effect of NAC on group I mGlus activation by DHPG and its potential role in the process of PD.

**Figure 3:**
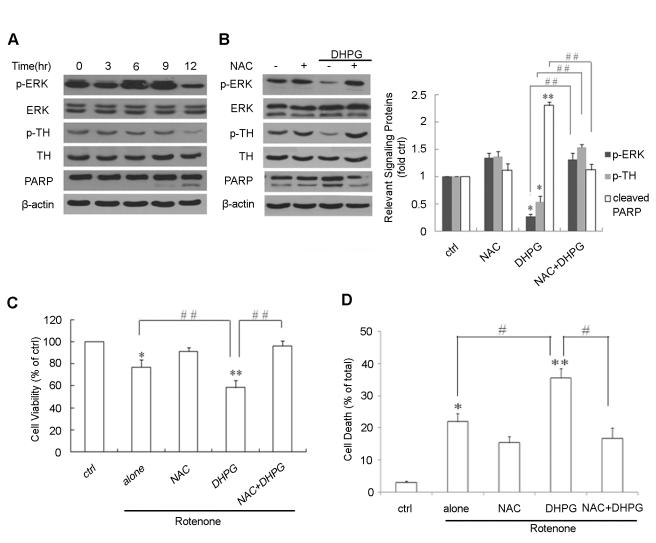
The effect of NAC on rotenone-induced toxicity in MN9D cells. (A) Cells were treated with 50 nM rotenone for different durations (0, 3, 6, 9 and 12 h). Representative Western blots show protein levels of phospho-ERK, phospho-TH and PARP cleavage. (B) Cells were incubated with NAC (5 mM) and/or stimulation with DHPG (100 µM), followed by rotenone together with NAC and DHPG incubation for 12 h. Western blotting was performed to determine phospho-ERK, phospho-TH and PARP cleavage (left). Densitometric analysis shows phospho-ERK, phospho-TH and cleaved PARP for each condition, compared with control cultures with rotenone alone (left columns) (right). (C) MTT assay for detecting cell viability. Cells were incubated with NAC (5 mM) and/or stimulation with DHPG (100 µM), followed by rotenone for 12 h. Non-treated cells were used as the control. Cell viability is expressed as a percentage of control cultures (left column). (D) NAC prohibited cell death induced by group I mGlus in rotenone-treated MN9D cells. Cells were incubated with NAC (5 mM) and/or stimulation with DHPG (100 µM) for 30 min, followed by rotenone with NAC ) and/or DHPG incubation for 12 h. Cell death was evaluated by Typan blue-exclusion assay, and expressed as a percentage of total cells. Cells without the treatment were as the control. *p<0.05 and **p<0.01 versus control; #p<0.05, # #p<0.01.

In vitro , we treated murine dopaminergic MN9D cells with the mitochondrial complex I inhibitor rotenone, an agent inducing apoptosis via oxidative stress involved in mitochondria-dependent pathways, for establishing a well-known neurotoxic model mimicking PD. The model was confirmed by Fig. 3A, exposure of cell cultures to rotenone decreased phospho-tyrosine hydroxylase (TH), an enzyme for the rate-limiting step in the biosynthesis of dopamine, and the phosphorylation form is active in cells [23]. PARP cleavage was increased and phosphorylation of ERK had declined by 12 h. Interestingly, the application of DHPG promoted the decrease in phospho-TH and further decreased phospho-ERK and the cleavage of PARP induced by rotenone treatment. However, these rotenone-induced effects can be inhibited by NAC pretreatment (Fig. 3B). We also tested the effect of caspase-3 in NAC protection against group I mGluRs-mediated cellular apoptosis. Under the experimental conditions, pretreatment of Z-DEVD-FMK, a specific caspase-3 inhibitor, with DHPG application also inhibited rotenone-induced cell death, suggesting that protection of NAC against group I mGlus-mediated cellular apoptosis is through caspase-3 activation (Fig. S2). Consistently, MTT assay showed that exposure of cells to rotenone decreased cell viability by 23%, and application of DHPG increased cell loss by an additional 18%. NAC inhibited rotenone-induced cell death in the presence of DHPG (Fig. 3C). The results were confirmed by using typan blue-exclusion assay to assess cell death (Fig.3D). Collectively, the data demonstrate that the exacerbation of rotenone-induced toxicity by group I mGlus activation in MN9D cells was alleviated by NAC treatment.

**Figure 4:**
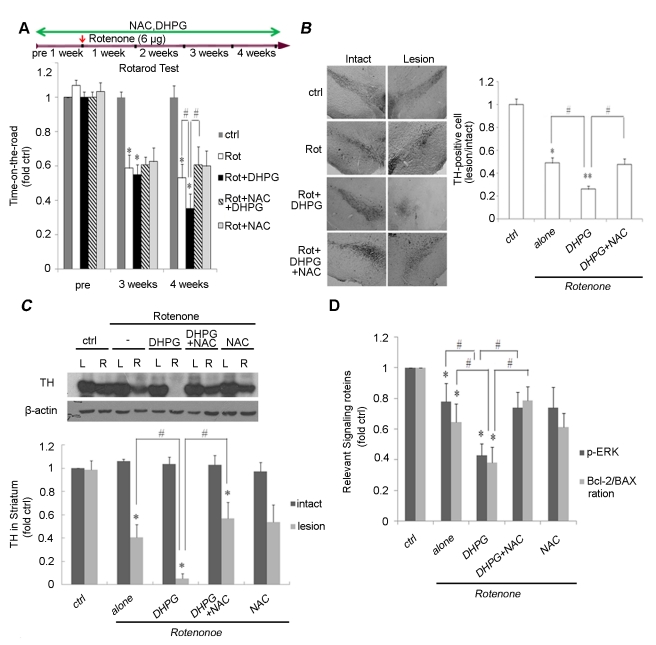
The effect of NAC on rotenone-induced rat model of PD. The treatment of rats with drugs is shown in the scheme (top). (A) Results of rotarod tests before ipsilateral rotenone injection, and 3 and 4 weeks after the injection of rotenone, shown as the length of time animals were able to remain on the rod at 5.r.p.m. Male Sprague-Dawley rats received ipsilateral rotenone (6 µg) injection after pretreatment with NAC (33 mg/kg/day) and DHPG (0.3 mg/kg/day) for 1 week and that the treatment has been continued until sacrifice. Time-on-the rod is expressed as a percentage of control before rotenone injection (left column). (B) Microphotographs of TH-positive neurons in rat substantia nigra by immunohistology (left). Magnification ×5 (Scale bar, 200 µm). Cell counts of TH-positive neurons in rat substantia nigra (n = 6) are also shown (right). (C) Representative Western blot showing the level of TH in the intact (left: L) and injected (right: R) striata of animals that received ipsilateral vehicle or rotenone injections. Densitometric analysis showing TH for each condition compared with control (left lane) (n = 10/group). (D) Densitometric analysis showing phospho-ERK and Bcl-2/Bax ratio in the striatum of animals for each condition compared with control (left lane). (E) NAC inhibited the oxidation of cellular GSH/GSSG (E_h_) induced by activated group I mGlus in rotenone-treated rat model of PD. *p<0.05 and **p<0.01 versus control; #p<0.05,# #p<0.01.

We determined if the protective effect of NAC in vitro could be corroborated in vivo, using a rotenone-induced rat model of PD. In the rotarod test, all groups of animals learned the task and were able to remain on the rod for 180 s at a speed of 5 rpm before rotenone injection. However, as shown in Fig. 4A, the time-on-the-rod was significantly reduced 3–4 weeks after rotenone administration, compared with vehicle-treated rats. Furthermore, DHPG-treated rats had a shorter time-on-the-rod, which was prolonged by NAC pretreatment, at 4 weeks after rotenone administration, indicating that NAC was able to alleviate the behavioral symptoms induced by group I mGlus activation in rotenone-treated rats. Changes in expression levels of TH in the substantia nigra and striatum were determined by immunohistology and Western blotting, respectively (Fig. 4B and C). A reduction in TH expression level was observed 4 weeks after rotenone administration, in line with the known sensitivity of nigro-striatal dopaminergic neurons to rotenone toxicity [24]–[25]. DHPG administration exacerbated the rotenone-induced decrease in TH in the lesioned substantia nigra and striatum, compared with the intact side. NAC treatment attenuated the decline in TH in rotenone-induced rats treated with DHPG. Furthermore, NAC treatment also attenuated the decline in ERK phosphorylation and the Bcl-2/Bax ratio mediated by DHPG treatment followed by rotenone treatmemt (Fig. 4D). Taken together, these results demonstrate that NAC suppressed the effects of group I mGlus activation by DHPG in a rotenone-induced rodent model of PD.

### The effect of NAC on the oxidative stress induced by activation of group I mGlus in glial C6 cells and rotenone-induced models of cells and rat of PD

**Figure 5:**
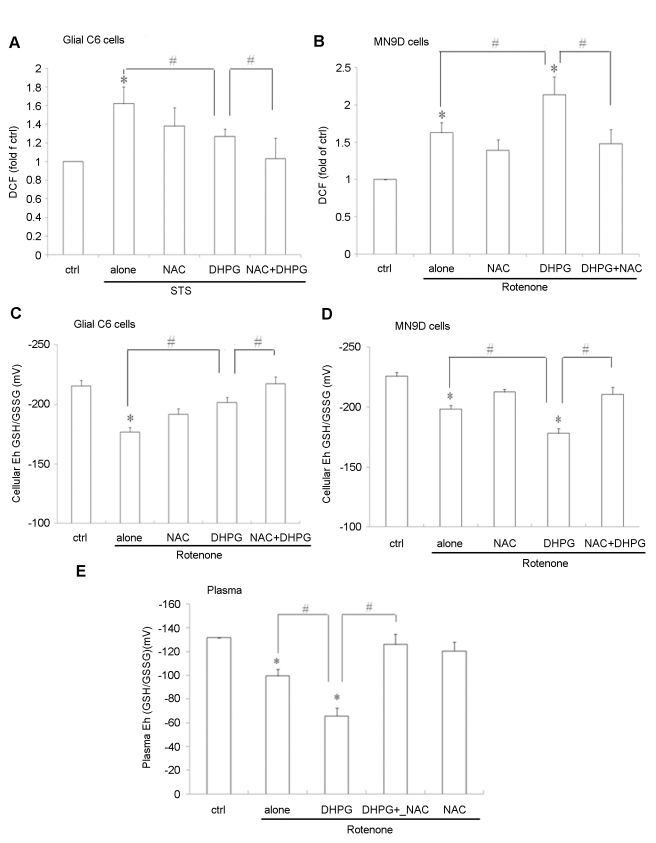
The effect of NAC on the oxidative stress induced by activation of group I mGlus in glial C6 cells and rotenone-treated models of cells and rat of PD. (A) NAC further prohibited the level of STS-induced ROS accompanied by activated group I mGlus in C6 cells. Cells were exposed to DHPG (100 µM, 30 min) after 5 mM NAC pretreatment, followed by STS exposure with NAC and DHPG incubation for 9 h and examined for cellular ROS measured by dichlorofluorescein (DCF) assay. Results are expressed as fold of control (left column). (B) NAC prohibited ROS production induced by activated group I mGlus in rotenone-treated MN9D cells. Cells were incubated with NAC (5 mM) and/or stimulation with DHPG (100 µM) for 30 min, followed by rotenone with NAC and /or DHPG for 9 h. Cells were detected for cellular ROS by DCF assay. The results are expressed as fold of control (left column). (C) NAC further prohibited the STS-induced oxidation of cellular GSH/GSSG (E_h_) accompanied by group I mGlus in glial C6 cells. Cells were exposed to DHPG (100 µM, 30 min) after 5 mM NAC pretreatment, followed by STS exposure with NAC and DHPG incubation for 9 h and examined for HPLC analysis of GSH and GSSG.. GSH/GSSG (E_h_) calculated from the GSH and GSSG concentrations using the Nernst equation. (D) NAC inhibited the oxidation of cellular GSH/GSSG (E_h_) induced by activated group I mGlus in rotenone-treated MN9D cells. Cells were incubated with NAC (5 mM) and/or stimulation with DHPG (100 µM) for 30 min, followed by rotenone with NAC and DHPG incubation for 9 h. Cells were then examined for GSH/GSSG (E_h_). (E) NAC inhibited the oxidation of cellular GSH/GSSG (E_h_) induced by activated group I mGlus in rotenone-treated rat model of PD. Cells without treatment were used as controls. *p<0.05 versus control; # p<0.05.

Antioxidant NAC, a precursor of GSH is known to scavenge ROS, and capable of reducing oxidized proteins [26]. Resting plasma GSH levels are lower in PD than in any other neurological condition [27]. To further examine whether NAC protected from apoptosis mediated by activation of group I mGlus through regulation of oxidative stress, we tested the effect of NAC on the intracellular ROS production and the redox potential of GSH/GSSG (E_h_) in the experimental models. The results showed that NAC further prohibited the level of STS-induced ROS (Fig. 5A) and oxidation of cellular GSH/GSSG (E_h_) (Fig. 5C) accompanied by activated group I mGlus in C6 cells. NAC also inhibited the level of ROS production (Fig. 5B) and the oxidation of cellular GSH/GSSG (E_h_) induced by activated group I mGlus in rotenone-treated MN9D cells (Fig. 5D). In addition, consistent with the report[26], plasma GSH/GSSG (E_h_) in rotenone-treated animals were observed to be oxidized, compared with vehicle-treated group. The plasma GSH/GSSG (E_h_) induced by DHPG was further oxidized compared to rotenone-treated animals, and NAC treatment efficiently inhibited the oxidation of GSH/GSSG (E_h_) caused by DHPG treatment (Fig. 5E). The data suggest that NAC might modulate group I mGlus-mediated signaling pathways to protect from cell apoptosis by reducing the oxidative stress.

## Discussion

This study investigated the relationship between group I mGlus activation and NAC in neuroprotection in glial C6 and MN9D cells, and in a rat model of PD. NAC demonstrated a protective effect on DHPG-mediated group I mGlus activation in the experimental models, via regulating group I mGlus-mediated ERK activity, whose activation functions in cell survival [7], [10]. In glial C6 cells, NAC promoted ERK activation induced by group I mGlus activation, however, in rotenone-treated MN9D cells, NAC complemented the inhibitory effect of group I mGlus activation on ERK phosphorylation. Moreover, in glial C6 cells, NAC enhanced the effects of group I mGlus activation via inhibition of cytochrome c release and the increases in the Bcl-2/Bax ratio and uncleaved PARP, further inhibiting STS-induced apoptosis. In a rotenone-treated MN9D cells and rat model of PD, treatment with NAC suppressed the effects of group I mGlus activation by DHPG to protect from cell death. These results revealed that NAC protected against apoptosis through regulating the activation of group I mGlus in both situations of neuroprotection and neurotoxicity, indicating that NAC might act as a regulator of group I mGlus-mediated activity to protect cell survival.

Activation of group I mGlus is involved in the survival of neurons via control of oxidative stress and regulation of intracellular GSH [4], [14]. However, how intracellular redox status controls group I mGlus activation remains unclear. Previous studies have shown that the pharmacologic modulation of mGlus can ameliorate motor abnormalities in experimental models of PD [27]–[28], and blockage of mGlu5 has shown a beneficial antidyskinetic effect in L-dopa-treated 1-methyl-4-phenyl-1,2,3,6-tetrahydropyridine **(** MPTP) **)** monkeys [29]. Moreover, oxidative stress to dopaminergic neurons is believed to be one of the causes of neurodegeneration in PD. In an animal model study, the antioxidants, NAC and L-2-oxothiazolidine-4-carboxylate (OTC) were suggested to prevent MPTP-induced apoptosis by suppressing JNK activation [30]. The results of our study indicate that antioxidant NAC regulates the activity of group I mGlus, as evidenced by changes in the activation of the receptor-mediated ERK pathway, cell viability and apoptosis. Furthermore, our results suggest a role for the regulation of group I mGlus activation by NAC in the process of PD, both in rotenone-induced cells and rat model. The modulation of NAC on group I mGlus activation might be through regulating intracellular GSH/GSSG (E_h_) to reduce the oxidative stress induced by rotenone and DHPG treatment (Fig. 5B and D). Indeed, the regulation of NAC on the level of ROS and GSH/GSSG (E_h_) with activation of group I mGlus was also found in glial C6 cells (Fig. 5A and C). In addition, NAC treatment alone also regulated the level of ROS and GSH/GSSG (E_h_) and had a protective effect in rotenone-induced MN9D cells and animal model; importantly, DHPG-induced oxidative stress, and subsequently, the relevant markers were found to be significantly affected by NAC treatment (Figs. 3 and 4), confirming that NAC can play a protective role in group I mGlus activation in the current experimental models. Taken together, these results reveal that NAC regulated the effects of group I mGlus activation on cell viability, indicating a possible role for intracellular redox status in the activation of group I mGlus associated with cell survival, especially in PD model.

.

The activation of group I mGlus has been shown to produce the relative neuroprotective or neurotoxic effects depending on different stimuli. For example, activation of these receptors downregulates nitric oxide-induced cysteine protease caspase-3 activity and prevents the induction of programmed cell death in rat hippocampal neurons [31]. On the other hand, selective antagonists of mGlu1 and mGlu5 can be neuroprotective, both in organotypic cultures subjected to 3-nitropropionic acid (3-NP) insult in vitro, and in a middle cerebral artery-occlusion model of ischemia in vivo [32]. The results of these studies suggest that the function of group I mGlus in cell viabilty varies among cell types, potentially controlled by differential signaling pathways. Indeed, we found that activation of group I mGlus inhibited STS-induced apoptosis in glial C6 cells and promoted ERK activation induced by group I mGlus activation (Figs. 1 and 2), however, in rotenone-treated MN9D cells and rat of PD, activation of group I mGlus promoted the cellular toxicity and inhibited ERK phosphorylation (Figs. 3 and 4). Whereas the effects of group I mGlus activation on cell apoptosis differed in those models, the protection of NAC against cellular toxicity with activated group I mGlus was found for both neuroprotective and neurotoxic situations, through modulation of receptor-mediated activity. Our study might provide a regulatory mechanism of group I mGlus activity in neuroprotection by NAC treatment.

In summary, this study showed that NAC was able to protect against apoptosis via modulating the activity of group I mGlus, in glial C6 and MN9D cells, as well as in a rotenone-treated rat model of PD. Additional studies are required to determine how NAC affects the activation of group I mGlus to protect cells from apoptosis in details. Although the mechanistic details of redox control of group I mGlus activation remain to be fully elucidated, this study has revealed a previously undescribed biological function for NAC in preventing neurotoxicity through regulating the activation of group I mGlus. These results contribute to the understanding of the pathogenesis of PD and could aid in the development of more effective ways to prevent dopaminergic neuronal death. These results, together with the distribution of group I mGlus in the brain, suggest that NAC might represent a new treatment for a variety of neurodegenerative diseases associated with group I mGlus.

## Materials and Methods

### Materials

DMEM/F-12 (1∶1; Gibco, USA), Dulbecco's modified eagle medium (DMEM) and fetal bovine serum were purchased from Hyclone (USA); U0126 (MAPK-ERK kinase (MEK) inhibitor), DHPG (a group I mGlus agonist); and (RS)-1-aminoindan-1,5-dicarboxylic acid (AIDA, a group I mGlus antagonist) were from Tocris Biosciences (UK); Z-DEVD-FMK (an irreversible and cell permeable inhibitor of caspase-3) was purchased from Becton, Dickinson and Company (BD, USA). NAC, buthionine sulfoximine (BSO) and staurosporine (STS) were obtained from Sigma (USA).

### Cell culture and Treatment

Rat C6 glial cells were obtained from the Institute of Cell Biology, Chinese Academy of Sciences (Shanghai, China) and maintained in complete medium (DMEM plus 10% fet al bovine serum and 1% penicillin/streptomycin) at 37°C in a 5% CO_2_ incubator. The MN9D dopaminergic neuronal cells were a generous gift from professor Hui Yang (Capital Medical University, Beijing, China) and were cultivated in complete medium (DMEM/F-12 plus 10% fet al bovine serum and 1% penicillin/streptomycin).

To stimulate or inhibit receptors, cell cultures were treated with either DHPG 100 µM for 5 min, or AIDA 10 µM for 30 min. To block the ERK pathway, cell cultures were preincubated with U0126 10 µM for 30 min before stimulation. STS and rotenone were prepared as 1 mM and 50 µM stock solutions, respectively, dissolved in dimethylsulfoxide (DMSO) and stored at −80°C until use. For treatments, STS and rotenone stock solutions were diluted with medium to 1 µM and 50 nM, respectively, and added to the cells.

### Protein analysis and Western blotting

After appropriate treatments, cells were lysed in lysis buffer (final concentration: 50 mM Tris (pH 8.0), 50 mM NaCl, 1% Nonidet P-40, 20 nM okadaic acid, 20 µM sodium orthovanadate, phosphatase inhibitor mixture (Pierce,USA), and protease inhibitor mixture (Pierce)). Protein concentrations were determined using a BCA assay kit (Pierce). Immunoblot analysis was performed using equal amounts of protein for each sample loaded onto 8% or 12% sodium dodecyl sulfate-polyacrylamide gels. Proteins were then transferred onto immobilon-P transfer membranes (Millipore, USA). Membranes were blocked for 1 h with 5% milk in TBST (20 mM Tris-HCl, pH 7.6, 137 mM NaCl, 0.05% Tween 20), followed by incubation with the indicated antibodies overnight. The blots were then rinsed with TBST and incubated with appropriate horseradish peroxidase (HRP)-conjugated secondary antibodies (Cell Signaling Technology, USA) for 1 h at room temperature, rinsed with TBST several times, and developed using a enhanced chemiluminescence (Applygen, China). The following antibodies were used for Western blotting: phospho-p38 mitogen-activated protein kinase (MAPK) (Thr180/Tyr182; #9211), p38 MAPK rabbit polyclonal antibody (#9212), PARP rabbit polyclonal antibody (#9542) and β-actin rabbit polyclonal antibody (#4967), cytochrome c rabbit polyclonal antibody (#4272), Bcl-2 rabbit polyclonal antibody (#2876), Bax rabbit polyclonal antibody (#2772), tyrosine hydroxylase (TH) rabbit polyclonal antibody (#2792), phospho-TH rabbit polyclonal antibody (Ser40; #2791), α-tubulin mouse polyclonal antibody (#3873) (all from Cell Signaling). Phospho-ERK1/2 rabbit monoclonal antibody (#05-797R) and ERK1/2 rabbit polyclonal antibody (#06-182) were from Millipore.

### Analysis of Cytosolic Cytochrome c Accumulation

Glial C6 cells were grown to 80% confluence in 10-cm diameter dishes. Cytochrome c release from mitochondria into the cytosol was measured by Western blot analysis. In brief, treated cells were washed twice with chilled PBS followed by the addition of 1 ml Mito-Cyto Buffer (Mitochondria/ Cytosol Kit, Applygen Technologies, China). After incubation on ice for 5 min, the cells were gently scraped off, ground 30–40 times and centrifuged at 800 *g* for 5 min at 4°C. The supernatants were further centrifuged at 12,000 *g* for 10 min at 4°C. The supernatant was collected as a cytosolic fraction and subjected to Western blot analysis, as described above.

### Cell viability, Apoptosis and Trypan blue-exclusion assay

The viability of MN9D cells was measured by 3-(4,5-dimethylthiazol-2-yl)-2,5-diphenyltetrazolium bromide (MTT) assay. Briefly, cells cultured on 96-well plates were incubated for 4 h at 37°C with 0.5 mg/ml MTT (Sigma). The formation of the formazan product, proportional to the number of viable cells, was measured colorimetrically at 490 nm, and the background was measured at 650nm after extraction with 100 µl DMSO (Sigma). Terminal deoxynucleotidyl transferase mediated dUTP-biotin nick-end labeling (TUNEL) staining was performed using a In Situ Cell Death Detection kit (Roche Applied Science, Germany). Briefly, glial C6 cells grown on glass coverslips were fixed with 4% paraformaldehyde and permeabilized with 0.1% Triton X-100, and processed for TUNEL staining (green/red). Hoechst stain (Sigma) was added to counterstain nuclei. Photomicrographs from 4–6 different fields in each coverslip were captured. Typically, 100–200 cells were analyzed to determine the number of TUNEL-positive (apoptotic) cells. Total numbers of neurons were identified by Hoechst staining (blue). Apoptotic cell numbers were presented as a percentage of TUNEL-positive cells in relation to total cell numbers. For trypan blue-exclusion assay, briefly, treated cells were stained with 4% trypan blue (Sigma, USA) solution for 5min, and assessed for cell death by counting viable (nonstained) and non-viable (blue) cells. Cell death is expressed as the percentage of non-viable cells to the total number of cells counted. Eight microscopic fields and approximately 300 cells were counted in trypan blue staining.

### Animals and Treatments

Male Sprague-Dawley rats (190–210 g) were used. Three rats were kept in each cage under standard laboratory conditions, with free access to standard laboratory food and tap water, a constant room temperature of 22°C, 50–60% humidity, and a natural day-night cycle. All procedures were approved by and performed in accordance with the Animal Care and Use Committee of Capital Medical University, Beijing, China (2006-0009). The investigation conformed to the Guide for the Care and Use of Laboratory Animals published by the US National Institutes of Health (NIH, 1996).

Rats were anesthetized by injection of chloral hydrate (150 mg/kg, intraperitoneally), and positioned in a stereotaxic apparatus (Lee. Y. M et al., 2008; Weng et al., 2007). Briefly, rats received a unilateral injection of (6 µg) rotenone in DMSO into the right medial forebrain bundle area, at a rate of 1 µl/min, using the coordinates 4.4-mm caudal and 1.1-mm lateral to the bregma, at a depth of 7.9 mm from the dural surface. After injection, the needle was left in place for an additional 10 min before slow retraction. Animals treated with DMSO alone were used as controls.

Rats were randomly divided into five groups. Control group of rats (n = 12) received vehicle only. One group of rats (n = 16) received ipsilateral rotenone (6 µg) injection. One group of rats (n = 16) received rotenone injection after DHPG administration (0.3 mg/kg/day, subcutaneously). One group of rats (n = 16) received rotenone (6 µg) injection after treatment with NAC (33 mg/kg/day) and DHPG treatment, and one group of rats ((n = 16) received rotenone injection after treatment with NAC. The treatment for the group has been continued until sacrifice. Some rats in the rotenone group died (20%) after rotenone injection, leaving10–12 animals per group. Rats were killed by decapitation 4 weeks after rotenone administration. Brains were removed and sliced using a tissue chopper. Samples from the various brain regions were collected by microdissection on a cold plate under a stereomicroscope. Total protein samples for Western blotting analysis were prepared by immediately freezing the tissues in dry ice and keeping them in a deep freeze until use. They were then homogenized in lysis buffer and total protein concentrations were determined by BCA assay.

### Behavioral Tests

The rotarod test was performed to evaluate the effects of DHPG and NAC on rotenone-induced Parkinsonian symptoms. All rats were tested at three time points: before administration, and 3 and 4 weeks after treatment. Only rats that completed all 4 weeks of the experiments were included in statistical analyses. The basic equipment consisted of a rotating roller (or rotarod) of the appropriate diameter (8 cm) for rats, a power source for turning the roller (control of rod speed), and four circular separators placed along the rod at suitable intervals to divide the roller into equal-sized compartments for simultaneous testing of four animals. Rats were placed on the rod and tested at a speed of 5 r.p.m for a maximum of 300 s. The length of time that each animal was able to stay on the rod was recorded.

### Immunohistochemistry

Animals were anesthetized with chloral hydrate and perfusion-fixed through the heart with 4% paraformaldehyde in 0.1 mM phosphate buffer pH 7.4. Brains were removed, post-fixed overnight in the same fixative and washed sequentially in buffered 15% and 30% sucrose until they sank. Sections were cut at 20 µm thickness using a freezing microtome, permeabilized for 15 min with PBS containing 0.5% Triton X-100 (Sigma), followed by 30 min incubation in methanol containing 3% H_2_O_2_ to quench endogenous peroxidase activity. Subsequently, sections were incubated for 30 min in PBS containing 5% BSA to block non-specific binding sites. For immunohistochemical localization of TH, we used a monoclonal anti-TH antibody at a dilution of 1∶10,000 (Sigma). Antibody exposure was performed overnight in a cold room, followed by 30 min incubation with a HRP-linked secondary antibody (Cell Signaling Technology), and the immunoreaction was visualized using diaminobenzidine tetrahydrochloride as a chromogen (Golden Bridge Biological Technology, China).

### Fluorescence-based Detection of Cellular ROS

Briefly, treated cells were loaded with 2′,7′-dichlorodihydrofluorescein diacetate (H_2_DCFDA, Sigma,USA) in loading medium (DMEM with 1% FBS) for 30 min. The medium were removed and cells were washed with phosphate-buffered saline (PBS). Fluorescence (488 nm excitation and 530 nm emission) was measured after 30 min in a microplate fluorometer (Thermo, USA). The value obtained for a blank well with only PBS was subtracted as the background optical density.

### High performance Liquid chromatography (HPLC) Analysis of GSH/GSSG Redox Potential (E_h_) of Cells and Plasma

The method has been described elsewhere ((Corinna et al., 2007, Smita et al, 2009). To measure cellular GSH/GSSG (E_h_), cells were washed once with 1 ml of PBS and immediately treated with 500 µl of ice-cold 5% (wt/vol) perchloric acid solution containing 0.2 M boric acid and10 µM γ-Glu-Glu and placed on ice. After 5 min, cells were scraped and transferred into microcentrifuge tubes. Samples were stored at −20°C until derivatization with iodoacetic acid and dansyl chloride. For HPLC analysis, derivatized samples were centrifuged, and 20 µl of the aqueous layer was applied to the Supelcosil LC-NH2 column (25 cm×4.6 mm; Supelco, Bellefonte, PA). Derivatives were separated with a sodium acetate gradient in methanol-water and detected by fluorescence. Concentrations of thiols and disulfides were determined by integration relative to the internal standard. The redox states of the GSH/GSSG (E_h_) were calculated from concentrations of GSH and GSSG in molar units with the following forms of the Nernst equation for pH 7.4: GSH/GSSG(E_h_) = −264+30log([GSSG]/[GSH]^2^).

To measure plasma GSH/GSSG (E_h_)_,_ derivatized blood samples were centrifuged, and 50 ml of the aqueous layer was applied to the Supercosil LC-NH2 column. Derivatives were separated with a sodium acetate gradient in methanol/water and detected by fluorescence. Concentrations of thiols and disulfides were determined by integration relative to the internal standard. Redox potentials (E_h_) of the GSH/GSSG, given in millivolts (mV), were calculated as the above.

### Statistical Analysis

All values are means±SEM. The significance of the difference between control and samples treated with various drugs was determined by one-way ANOVA followed by the post hoc least significant difference test. Differences were considered significant at p<0.05.

## Supporting Information

Figure S1 **Caspase-3 is involved in NAC protection from group I mGlus-mediated apoptosis in STS-treated glial C6 cells.** (A) Cells were exposed to NAC (5 mM ) and DHPG (100 µM, 30 min) after pretreatment with the caspase-3 inhibitor Z-DEVD-FMK (50 µM, 1h), followed by STS exposure for 12 h. The effect of caspase-3 was examined in TUNEL staining experiments. Magnification ×400 (n = 4–6 microscopic fields). (B) Densitometric analysis of TUNEL-positive cells, expressed as a percentage of total cells. #p<0.05.
(TIF)

Figure S2 **Caspase-3 is involved in NAC protection from group I mGlus-mediated apoptosis in rotenone-treated MN9D cells.** (A) Cells were exposed to NAC (5 mM ) and DHPG (100 µM, 30 min) after pretreatment with the caspase-3 inhibitor Z-DEVD-FMK (50 µM, 1h), followed by rotenone exposure for 12 h. The effect of caspase-3 was examined in TUNEL staining experiments. Magnification ×400 (n = 4–6 microscopic fields). (B) Densitometric analysis of TUNEL-positive cells, expressed as a percentage of total cells. #p<0.05.
(TIF)
