# Supplementary figures and images for: N-acetylcysteine Protects against Apoptosis through Modulation of Group I Metabotropic Glutamate Receptor Activity
Source: PLoS One. 2012 Mar 19;7(3):e32503. doi: 10.1371/journal.pone.0032503 (PMC3307713; doi:10.1371/journal.pone.0032503)

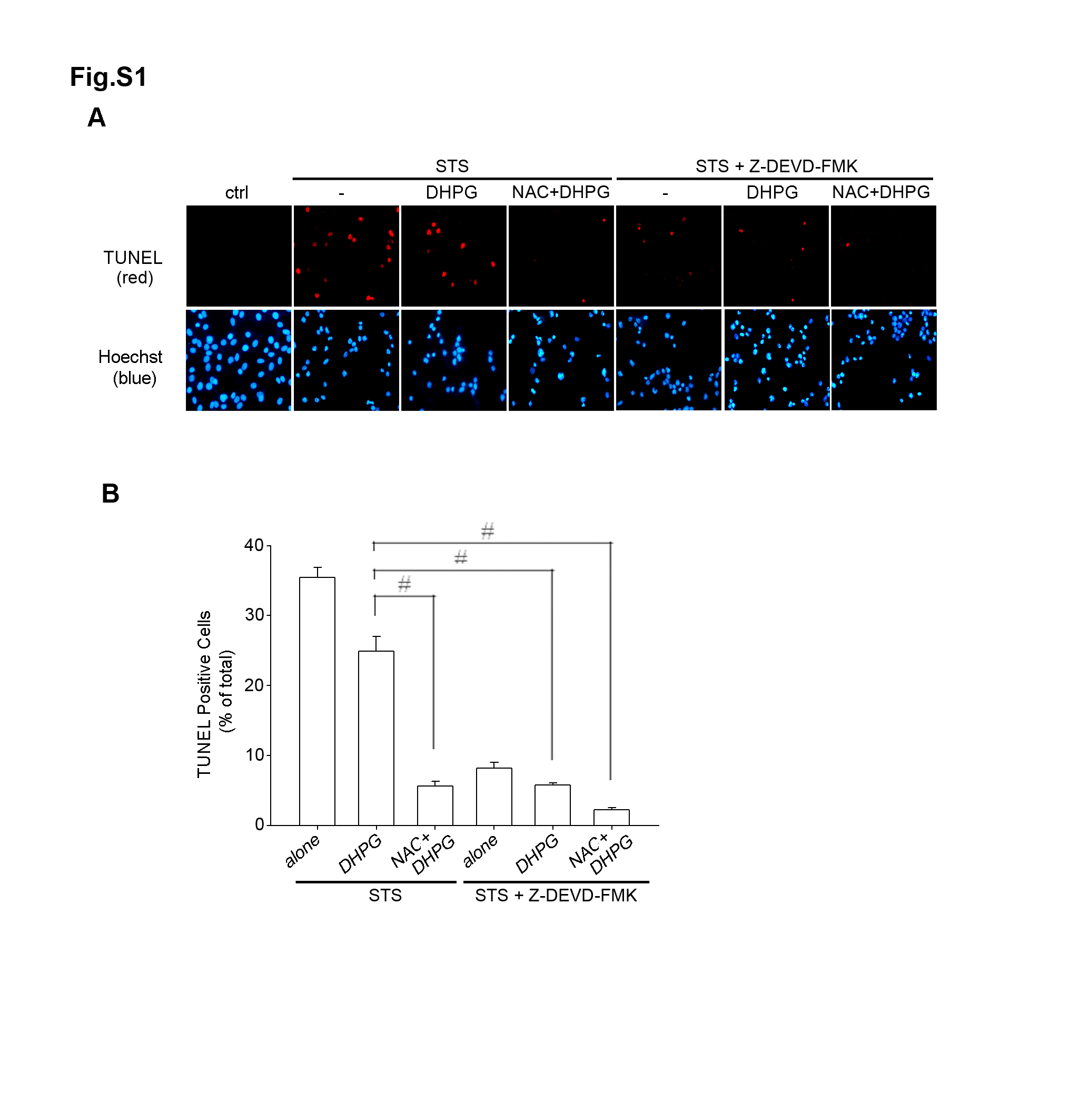

Supplement: Figure S1 — Caspase-3 is involved in NAC protection from group I mGlus-mediated apoptosis in STS-treated glial C6 cells. (A) Cells were exposed to NAC (5 mM ) and DHPG (100 µM, 30 min) after pretreatment with the caspase-3 inhibitor Z-DEVD-FMK (50 µM, 1h), followed by STS exposure for 12 h. The effect of caspase-3 was examined in TUNEL staining experiments. Magnification ×400 (n = 4–6 microscopic fields). (B) Densitometric analysis of TUNEL-positive cells, expressed as a percentage of total cells. #p<0.05. (TIF) [file pone.0032503.s001.tif]

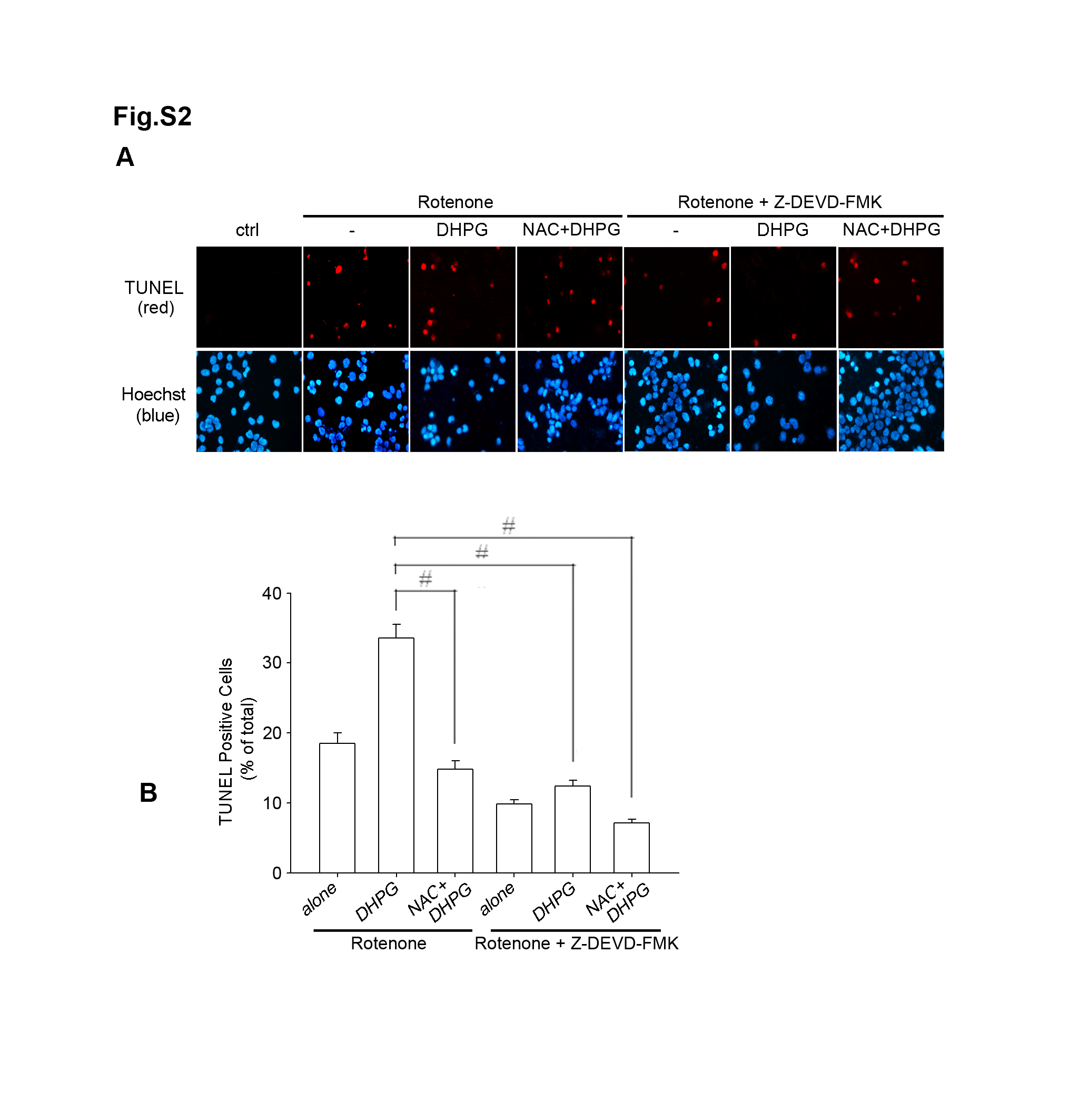

Supplement: Figure S2 — Caspase-3 is involved in NAC protection from group I mGlus-mediated apoptosis in rotenone-treated MN9D cells. (A) Cells were exposed to NAC (5 mM ) and DHPG (100 µM, 30 min) after pretreatment with the caspase-3 inhibitor Z-DEVD-FMK (50 µM, 1h), followed by rotenone exposure for 12 h. The effect of caspase-3 was examined in TUNEL staining experiments. Magnification ×400 (n = 4–6 microscopic fields). (B) Densitometric analysis of TUNEL-positive cells, expressed as a percentage of total cells. #p<0.05. (TIF) [file pone.0032503.s002.tif]
